# Retraction

**DOI:** 10.1002/cam4.5823

**Published:** 2023-04-12

## Abstract

Gao Y, Wang F, Zhang L, et al. LINC00311 promotes cancer stem‐like properties by targeting miR‐330‐5p/TLR4 pathway in human papillary thyroid cancer. *Cancer Med*. 2020; 9: 1515–1528. https://doi.org/10.1002/cam4.2815.

The above article, published online on 02 January 2020 in Wiley Online Library (wileyonlinelibrary.com), has been retracted by the journal's Editor‐in‐Chief, Dr Stephen Tait, and John Wiley & Sons Ltd. The retraction has been agreed due to concerns regarding image manipulation, which were brought to the journal's attention by a third party. A detailed investigation confirmed substantial inconsistencies. As a result, the journal no longer has confidence in the results and conclusions reported.

Gao Y, Wang F, Zhang L, et al. LINC00311 promotes cancer stem‐like properties by targeting miR‐330‐5p/TLR4 pathway in human papillary thyroid cancer. *Cancer Med*. 2020; 9: 1515–1528. https://doi.org/10.1002/cam4.2815.

The above article, published online on 02 January 2020 in Wiley Online Library (wileyonlinelibrary.com), has been retracted by the journal's Editor‐in‐Chief, Dr Stephen Tait, and John Wiley & Sons Ltd. The retraction has been agreed due to concerns regarding image manipulation, which were brought to the journal's attention by a third party. A detailed investigation confirmed substantial inconsistencies. As a result, the journal no longer has confidence in the results and conclusions reported.

